# Development of the Adjusted Wind Chill Equivalent Temperature (AWCET) for cold mortality assessment across a subtropical city: validation and comparison with a spatially-controlled time-stratified approach

**DOI:** 10.1186/s12889-019-7612-5

**Published:** 2019-10-15

**Authors:** Hung Chak Ho, Man Sing Wong, Sawaid Abbas, Rui Zhu

**Affiliations:** 1 Department of Urban Planning and Design, The University of Hong Kong, Hong Kong, Hong Kong; 2 Department of Land Surveying and Geo-informatics, The Hong Kong Polytechnic University, Hong Kong, Hong Kong; 3 Research Institute for Sustainable Urban Development, The Hong Kong Polytechnic University, Hong Kong, Hong Kong; 4 Senseable City Laboratory, Singapore-MIT Alliance for Research and Technology, Singapore, Singapore

**Keywords:** Cold stress, Mortality assessment, Biometeorological index, Wind chill, Subtropical

## Abstract

**Background:**

Global warming has reduced the adaptability of the people living in subtropical regions to cope up with cold stress due to lengthening of hot days and shortening of transition period from hot to cold weather. However, existing studies on measuring cold stress are based on biometeorological indices designed for temperate regions. This may overestimate the impact of wind chill on mortality risk in subtropical cities.

**Methods:**

This study developed an Adjusted Wind Chill Equivalent Temperature (AWCET) index. A spatially-controlled time-stratified approach was applied to evaluate the ability of AWCET for estimating cold mortality in subtropical cities, based on a mortality dataset (2008–2012) in Hong Kong.

**Results:**

The use of AWCET could indicate increase in all-cause, cardiovascular, respiratory, and cancer-related mortality risk during the days with average temperature < = 1st [11.0 °C], <= 3rd [12.6 °C] and < = 5th [13.4 °C] percentiles. The results were stable and consistent based on both log-linear and curve-linear relationships between AWCET and mortality risk. AWCET was also compared with the New Wind Chill Equivalent Temperature (NWCET) designed for temperate regions, and has found that higher magnitude of mortality risk would be found when using AWCET for assessing all-cause and cause-specific mortality in Hong Kong, for days with average temperature < = 1st, <= 3rd and < = 5th percentiles.

**Conclusions:**

AWCET is validated to be effective to access cold mortality in the context of subtropical cities. The use of AWCET may enhance the cold weather warning system in subtropical cities, as a supplementary tool to help demonstrating small administrative-level perceived temperature with volunteered geographic information.

## Background

Global warming has reduced the adaptability of cold stress among urban population, especially to people in subtropical cities [1]. Previous studies have pointed out that subtropical and tropical cities can have higher mortality risk from cold stress than heat [2, 3], due to a lack of adaptation. To evaluate the mortality risk caused by extreme cold event, estimation of temperature-mortality relationship is a common approach to analyze the excessive mortality caused by the decreasing temperature [4]. However, air temperature may not be the solely appropriate indicator for measuring the cold stress, because this is normally shown as the ambient temperature without inclusion of biometeorological factors.

To tackle the problem above, biometeorological indices have been developed and used to assess cold stress. For example, physiological equivalent temperature (PET) and minimum net effective temperature (NET) have been applied to evaluate temperature-mortality relationship across various cities in an all-seasons scenario [5, 6]. However, these biometeorological indices have a problem in nature. Specifically, PET and NET were designed based on an all-seasons scenario with components related to humidity and solar radiation. However, a recent article has illustrated that humidity and solar radiation were weakly and inconsistently associated with winter mortality in subtropical cities [7]. In addition, some components of weather information such as solar radiation are difficult to be implemented to a district-level microclimate advisory system. Therefore, using such biometeorological indices designed for an all-seasons scenario may not be appropriate to evaluate cold stress across a city, especially to the city with a subtropical weather.

Based on the limitation above, previous studies have also developed biometeorological indices specifically for cold weather, such as Wind chill index and the New Wind Chill Equivalent Temperature (NWCET) [8, 9]. These biometeorological indices have been using as Government-based measures to monitor cold stress in countries located in temperate regions. However, these indices were designed based on the cold scenario in temperate regions, which may not be applicable in subtropical cities. Even these biometeorological indices have been verified by health data, the validation has only been conducted into the extreme case in temperate regions [10]. Therefore, adjustment of such indices is critical since the adjusted indices may enhance the applications for cold mortality assessment in subtropical cities. Furthermore, several government agencies pointed out that such indices might overstate the wind chill effect on health in subtropical cities, based on climatic perspectives [11]. This indicated that the adjustment of winter-based biometeorological indices for applications should include a modification of wind chill effects.

This study proposes an Adjusted Wind Chill Equivalent Temperature (AWCET) based on NWCET, which is more suitable for cold mortality assessment in subtropical regions. This AWCET was developed and implemented based on the scenario in Hong Kong. Located in the subtropical region, Hong Kong has found higher mortality risk during winter than summer, due to low adaptability of local population [1]. Using the daily minimum net effective temperature (NET) considering temperature, humidity and wind speed as measure, the Hong Kong Observatory (HKO) has found that the mean mortality can be increased by approximately 1.3-fold per unit decrease in NET, when NET is below 14 in Hong Kong [6]. Another publication from the HKO [12] also found that winter mortality (Nov - Mar) between 1968 and 1995 was significantly higher than summer mortality (May - Sept), and this result led to the government-based decision to establish the cold weather warning for local population in Hong Kong. During an extreme cold event with temperature lower than the 1st percentile, relative risk of non-accidental mortality can be 17% higher than the other days for a 14-days period [13]. Specifically, older people and persons with cardiorespiratory diseases were more vulnerable during the cold events [14].

As an aging society, cold stress has been a serious concern of general population [14]. The public concern has been addressed by a population-based study [15], in which 95.7% respondents have reported to be aware of the cold warning from the Hong Kong Observatory, indicating that cold stress is a known problem increasing perceived risk of local population. However, despite the fact that Hong Kong has already had NET for assessing thermal discomfort in Hong Kong, the use of NET may not be the best for evaluating cold stress, because the design of NET itself was based on an all-seasons scenario as stated in previous sections. Thus, an implementation of a biometeorological index specifically for cold mortality assessment in subtropical city would be more beneficial to the public.

Therefore, the objective of this study is to develop AWCET particularly for the cold mortality assessment across Hong Kong. The AWCET was also evaluated by a spatially-controlled time-stratified approach for cold mortality assessment, and was compared with the performance of NWCET, in order to demonstrate the abilities of AWCET in predicting cold effects in a subtropical city. In conclusion, the results drawn from the study could be applied elsewhere in the subtropical regions with a similar climate.

## Data and methods

### Data collection

Mortality data between 2008 and 2012 for each decedent of Hong Kong were used. This mortality dataset included the 1) date of death of each decedent, 2) age, 3) gender, 4) occupation, 5) marital status, 6) location of residence, and 7) cause of death. Location of residence of each decedent of this mortality dataset was registered based on the tertiary planning unit (TPU), which divides Hong Kong in 287 sub-districts. Cause of death of each decedent was record based on the 10th revision of the International Statistical Classification of Diseases and Related Health Problems (ICD-10).

Daily weather information including average temperature, average relative humidity (RH) and average wind speed were obtained from the Hong Kong Observatory recorded at the meteorological station located at the headquarters. Daily averages of respirable suspended particulates (RSP), nitrogen oxides (NO_X_) and ground-level ozone (O_3_) were collected from the Environmental Protection Department (EPD) of Hong Kong based on the following seven monitoring stations: Central Western, Sham Shui Po, Sha Tin, Tai Po, Tsuen Wan, Kwai Chung, and Tap Mun. Note that RSP defined by EPD are “the particulate matters with aerodynamic diameter less than or equal to 10 micrometers”, which is a composite of coarse particulate matters (PM_10–2.5_) and fine particulate matters (PM_2.5_).

Percent of low-education population (*low education %*), and percent of the population speaking foreign languages (*foreign languages %*) in each TPU were also collected based on the 2006 Hong Kong census data to represent the socioeconomic disparity across the city. Note that *low education %* in this study was defined as the percentage of people with a primary school education or less, and *foreign languages %* was defined by the percentage of persons whose native language is not Cantonese.

Average of Normalized Difference Vegetation Index (NDVI) for each TPU was also calculated to represent vegetation cover and urban/rural difference across the city, with a range from – 1 to 1 [16, 17]. The NDVI derived from an IKONOS multispectral image resampled to 15 m resolution, in which lower values indicated potentially more urbanized areas with less vegetation, while higher values represented vegetated or densely vegetated areas.

### The adjusted wind chill equivalent temperature (AWCET)

The Adjusted Wind Chill Equivalent Temperature (AWCET) was developed based on the New Wind Chill Equivalent Temperature (NWCET) written as follows:

$$ NWCET=13.12+0.6215\times Ta-11.37\ {WV}^{0.16}+0.3965\times Ta\times {WV}^{0.16} $$

where Ta is the air temperature in °C and WV is the wind velocity in km/hour.

This original NWCET was designated for a cold scenario for temperate regions [9] and was evaluated to be relevant to human discomfort and health risk during wintertime [10]. In addition, NWCET has been used as the governmental measure of winter perceived temperature in the United States and Canada.

In the context of subtropical cities (e.g. Hong Kong), wind chill effect may be much lower than in the temperate regions. High-density built-up environment with compact settings of the subtropical cities can reduce the wind chill effect. Therefore, this study initially hypothesized that wind chill effect in subtropical cities may be 3 to 4 times weaker than the temperate regions, as a result, the equation for AWCET is designed as the follows:

$$ AWCET=13.12+0.6215\times Ta-11.37\ {\left(\frac{WV}{3.6}\right)}^{0.16}+0.3965\times Ta\times {\left(\frac{WV}{3.6}\right)}^{0.16} $$

where Ta is the air temperature in °C and WV is the wind velocity in km/hour.

Based on the adjusted equation, AWCET has only included approximately 28% of wind chill effects from the original formula in perceived temperature estimation. The use of 1/3.6 is also because of the ratio for interchanging/conversion of unit from km/hour to m/s for meteorological applications.

### Spatially-controlled time-stratified approach

This study applied a spatially-controlled time-stratified approach to validate the ability of AWCET in cold mortality assessment. To evaluate the stability of AWCET and to reduce the bias from selection of control groups, three sets of cases were selected and were compared with four sets of controls, separately. The following were the cases: 1) decedents died from days with average temperature < =5th percentile; 2) decedents died from days with average temperature < =3rd percentile; and 3) decedents died from days with average temperature < =1st percentile. The following were the controls: 1) decedents from the same day and same weekday for the 4 weeks before; 2) decedents from the same day and same weekday for the 8 weeks before; 3) decedents from the same day and same weekday for the 4 weeks after; 4) decedents the same day and same weekday for the 8 weeks after. A binomial regression was applied to estimate the mortality risk contributed by a 1 °C decrease in AWCET as follows:

$$ case\left(1,0\right)\sim {\beta}_0+{\beta}_1\times \left[\left(-1\right)\times {AWCET}^n\right]+{\beta}_2\times RH+{\beta}_3\times RSP+{\beta}_4\times {NO}_x+{\beta}_5\times {O}_3+{\beta}_6\times NDVI+{\beta}_7\times low\  education\%+{\beta}_8\times foreign\ languages\%+{\beta}_9\times unemployed\ \left(1,0\right)+{\beta}_{10}\times unmarried\left(1,0\right)+{\beta}_{11}\times age+{\beta}_{12}\times male\ \left(1,0\right)+{\beta}_{13}\times DOW $$

where case (1,0) represents decedents as cases or controls; RH is a confounder controlling for humidity; RSP, NO_x_ and O_3_ are the confounders controlling for air quality; NDVI, *low education%* and *foreign languages %* are neighbourhood-based confounders spatially controlling for urban/rural difference and socioeconomic disparity; unemployed (1,0) is a binary confounder with “1” as unemployed and “0” as employed; unmarried (1,0) is a binary confounder with “1” as unmarried and “0” as married; age is a continuing variable controlling for aging effect; male (1,0) is a binary confounder controlling for gender effect with “1” as male and “0” as female; and DOW is a continuous variable of the day of week controlling for weekday/weekend effect. In this study, we controlled the effect of each air pollutant separately [18, 19] instead of a summarized function describing the effects of three air pollutants [20], since previous studies have noted that various air pollutants could have different but significant impacts directly on daily mortality in Hong Kong [21, 22]. In addition, n is the exponent for linearity and nonlinearity. This study separately applied 1 and 2 for n to the regression for the assessment of both log-linear effect and curve-linear effect, as these effects have been used in previous studies to estimate the association between weather and health [23–27].

This study repeated the analyses for the following four groups of decedents: 1) all-cause deaths, 2) cardiovascular deaths (ICD-10 I00-I99), 3) respiratory deaths (ICD-10 J00-J99), and 4) cancer-related mortality (ICD-10 C00-C97). Particularly, specific causes of death used in this study were identified to be associated with extreme cold weather in previous studies [2, 3, 6–14, 28–30].

Odds ratio (OR) was reported with 95% confidence intervals for the evaluation of effect of a 1 °C decrease in AWCET on mortality risk. All analyses were performed with the *glm2* package of *R* software. Since missing information of death date as well as the location of residence were completely at random, a listwise deletion was applied to reduce the statistical bias.

### Performance comparison between AWCET and NWCET

Based on the model above, we repeated the regression above to evaluate the impact of NWCET on cold mortality, by considering log-linear effect on all-cause mortality, cardiovascular mortality, respiratory mortality, and cancer-related mortality. The OR between AWCET and NWCET was compared. If an OR and its 95% confidence intervals for AWCET were higher than the result for NWCET, it was determined as a result with significantly higher magnitude of mortality risk. If an OR and its 95% confidence intervals from a result for AWCET were lower than that for NWCET, it was a lower magnitude of mortality risk. Based on the difference in magnitude of mortality risk, performance of using AWCET or NWCET as biometeorological index for cold assessment was evaluated and compared.

## Results

### Data summary

Based on the weather information obtained from the Hong Kong Observatory, the 5th percentile of average temperature between 2008 and 2012 was approximately 13.4 °C. In addition, the 3rd percentile of average temperature was approximately 12.6 °C, and the 1st percentile of average temperature was approximately 11.0 °C. There were 92 days with average temperature < = 5th percentile between 2008 and 2012, including 55 days <=3rd percentile and 19 days <= 1st percentile. After missing data exclusion based on listwise deletion, our analytic dataset included 10,235 deaths from days with average temperature < = 5th percentile between 2008 and 2012, including 2465 deaths from cardiovascular diseases, 2463 deaths from respiratory diseases, and 3066 deaths from cancer. Specifically, there were 6147 deaths from days with average temperature < = 3rd percentile between 2008 and 2012, including 1504 deaths from cardiovascular diseases, 1476 deaths from respiratory diseases, and 1829 deaths from cancer. There were 2108 deaths from days with average temperature < = 1st percentile between 2008 and 2012, including 507 deaths from cardiovascular diseases, 485 deaths from respiratory diseases, and 647 deaths from cancer.

### Assessment of all-cause mortality

The results have shown that a 1 °C decrease in AWCET can significant indicate cold mortality risk in Hong Kong. For a day with average temperature < =5th percentile, AWCET can indicate higher risk of all-cause mortality during the colder day, regardless the changes of control groups. Considering the log-linear effect, a 1 °C decrease in AWCET can result to OR of 1.706 [1.682, 1.731], 1.794 [1.770, 1.819], 1.705 [1.679, 1.732], and 1.825 [1.798, 1.853] (Table 1), controlling for the effects of sociodemographic characteristics, neighbourhood disparity, urban/rural difference, air quality, and humidity as well as the weekday/weekend effect. Considering the curve-linear effect, a 1 °C decrease in AWCET can result to OR of 1.020 [1.019, 1.020], 1.021 [1.021, 1.022], 1.020 [1.019, 1.020], and 1.022 [1.022, 1.023].

**Table 1:** Odds ratios (OR) for assessment of AWCET validation

Temperature thresholds for cases		Controls: (Same day and same weekday for 4 weeks before)		Controls: (Same day and same weekday for 8 weeks before)		Controls: (Same day and same weekday for 4 weeks after)		Controls: (Same day and same weekday for 8 weeks after)	
		Log-linear	Curve-linear	Log-linear	Curve-linear	Log-linear	Curve-linear	Log-linear	Curve-linear
All-cause mortality	Average temperature < = 5th percentile	1.706 [1.682, 1.731]	1.020 [1.019, 1.020]	1.794 [1.770, 1.819]	1.021 [1.021, 1.022]	1.705 [1.679, 1.732]	1.020 [1.019, 1.020]	1.825 [1.798, 1.853]	1.022 [1.022, 1.023]
	Average temperature < = 3rd percentile	1.877 [1.837, 1.919]	1.024 [1.022, 1.025]	2.011 [1.968, 2.054]	1.026 [1.025, 1.027]	1.937 [1.892, 1.983]	1.025 [1.024, 1.026]	2.108 [2.059, 2.157]	1.028 [1.027, 1.029]
	Average temperature < = 1st percentile	2.463 [2.303, 2.634]	1.038 [1.035, 1.041]	2.768 [2.588, 2.960]	1.043 [1.040, 1.046]	2.514 [2.356, 2.683]	1.038 [1.035, 1.041]	2.947 [2.764, 3.142]	1.045 [1.042, 1.048]
Cardiovascular mortality	Average temperature < = 5th percentile	1.718 [1.668, 1.770]	1.020 [1.09, 1.021]	1.802 [1.752, 1.854]	1.022 [1.020, 1.023]	1.704 [1.650, 1.758]	1.020 [1.019, 1.021]	1.817 [1.762, 1.875]	1.022 [1.021, 1.023]
	Average temperature < = 3rd percentile	1.880 [1.798, 1.967]	1.024 [1.022, 1.026]	2.011 [1.924, 2.101]	1.026 [1.024, 1.028]	1.932 [1.841, 2.028]	1.025 [1.023, 1.027]	2.092 [1.995, 2.193]	1.028 [1.026, 1.030]
	Average temperature < = 1st percentile	2.595 [2.233, 3.015]	1.040 [1.033, 1.047]	2.959 [2.544, 3.440]	1.046 [1.039, 1.053]	2.564 [2.232, 2.946]	1.039 [1.032, 1.045]	2.961 [2.584, 3.393]	1.045 [1.039, 1.051]
Respiratory mortality	Average temperature < = 5th percentile	1.691 [1.642, 1.741]	1.019 [1.018, 1.020]	1.771 [1.723, 1.821]	1.021 [1.020, 1.022]	1.719 [1.665, 1.774]	1.020 [1.019, 1.021]	1.838 [1.782, 1.896]	1.023 [1.021, 1.024]
	Average temperature < = 3rd percentile	1.853 [1.773, 1.938]	1.023, 1.021, 1.025]	1.983 [1.898, 2.071]	1.026 [1.024, 1.028]	1.939 [1.849, 2.034]	1.025 [1.023, 1.027]	2.107 [2.010, 2.209]	1.028 [1.026, 1.030]
	Average temperature < = 1st percentile	2.438 [2.125, 2.798]	1.038 [1.031, 1.044]	2.756 [2.404, 3.160]	1.043 [1.036, 1.049]	2.501 [2.196, 2.849]	1.037 [1.031, 1.043]	2.945 [2.590, 3.350]	1.045 [1.39, 1.051]
Cancer-related mortality	Average temperature < = 5th percentile	1.708 [1.665, 1.753]	1.020 [1.019, 1.021]	1.803 [1.760, 1.848]	1.022 [1.021, 1.023]	1.706 [1.659, 1.755]	1.020 [1.019, 1.021]	1.829 [1.780, 1.880]	1.022 [1.021, 1.023]
	Average temperature < = 3rd percentile	1.887 [1.813, 1.964]	1.024 [1.022, 1.026]	2.020 [1.943, 2.101]	1.026 [1.025, 1.028]	1.936 [1.854, 2.022]	1.025 [1.024, 1.027]	2.113 [2.025, 2.205]	1.029 [1.027, 1.030]
	Average temperature < = 1st percentile	2.337 [2.087, 2.615]	1.036 [1.030, 1.041]	2.594 [2.318, 2.902]	1.040 [1.035, 1.046]	2.404 [2.144, 2.697]	1.036 [1.031, 1.041]	2.845 [2.539, 3.187]	1.044 [1.038, 1.049]

The results remained stable when comparing with the days with lower temperatures. For a day with average temperature < =3rd percentile, cold morality risk was significantly higher than from a day with average temperature < =5th percentile. Considering the log-linear effect, a 1 °C decrease in AWCET in days with average temperature < =3rd percentile can result to OR of 1.877 [1.837, 1.919], 2.011 [1.968, 2.054], 1.937 [1.892, 1.983], and 2.108 [2.059, 2.157], controlling for all factors. For a day with average temperature < =1st percentile, cold morality risk was significantly higher than from days with average temperature < =5th percentile or < 3rd percentile. Considering the log-linear effect, a 1 °C decrease in AWCET in days with average temperature < =1st percentile can result to OR of 2.463 [2.303, 2.634], 2.768 [2.588, 2.960], 2.514 [2.356, 2.683], and 2.947 [2.764, 3.142]. Similar patterns were also found for the results considering the curve-linear effect.

### Assessment of cardiovascular mortality

The use of AWCET is consistently stable when it was applying to assess the excess mortality caused by cardiovascular diseases during an extreme cold event. Considering the log-linear effect, for a day with average temperature < =5th percentile, a 1 °C decrease in AWCET can result to OR of 1.718 [1.668, 1.770], 1.802 [1.752, 1.854], 1.704 [1.650, 1.758], and 1.817 [1.762, 1.875], controlling for the effects of sociodemographic characteristics, neighbourhood disparity, urban/rural difference, air quality, and humidity as well as the weekday/weekend effect (Table 1). Days with average temperature < =3rd percentile had higher mortality risk than days with average temperature < =5th percentile, in which considering the log-linear effect, a 1 °C decrease in AWCET in days with average temperature < =3rd percentile can result to OR of 1.880 [1.798, 1.967], 2.011 [1.924, 2.101], 1.932 [1.841, 2.028], and 2.092 [1.995, 2.193], controlling for all factors. Days with average temperature < =1st percentile also had higher mortality risk than days with average temperature < =3rd percentile, in which considering the log-effect effect, a 1 °C decrease in AWCET in days with average temperature < =1st percentile can result to OR of 2.595 [2.233, 3.015], 2.959 [2.544, 3.440], 2.564 [2.232, 2.946], and 2.961 [2.584, 3.393]. Similar patterns were also found for the results considering the curve-linear effect.

### Assessment of respiratory mortality

Similar observations can be found for the assessment of respiratory mortality (Table 1). For days with average temperature < =5th percentile, considering the log-linear effect, a 1 °C decrease in AWCET can result to OR for respiratory mortality of 1.691 [1.642, 1.741], 1.771 [1.723, 1.821], 1.719 [1.665, 1.774], and 1.838 [1.782, 1.896], controlling for the effects of sociodemographic characteristics, neighbourhood disparity, urban/rural difference, air quality, and humidity as well as the weekday/weekend effect. For days with average temperature < =3rd percentile, considering the log-linear effect, a 1 °C decrease in AWCET can result to OR for respiratory mortality of 1.853 [1.773, 1.938], 1.983 [1.898, 2.071], 1.939 [1.849, 2.034], and 2.107 [2.010, 2.209], controlling for the effects of sociodemographic characteristics, neighbourhood disparity, urban/rural difference, air quality, and humidity as well as the weekday/weekend effect. The OR for days with average temperature < =3rd percentile were significantly higher than the OR for days with average temperature < =5th percentile. In addition, for days with average temperature < =1st percentile, considering the log-linear effect, a 1 °C decrease in AWCET can result to OR for respiratory mortality of 2.438 [2.125, 2.798], 2.756 [2.404, 3.160], 2.501 [2.196, 2.849], and 2.945 [2.590, 3.350], controlling for all factors. The OR for days with average temperature < =1st percentile were significantly higher than the OR for days with average temperature < =3rd percentile and < =5th percentile. Similar patterns were also found for the results considering the curve-linear effect.

### Assessment of cancer-related mortality

The consistency of using AWCET for cold mortality assessment was not only found for all-cause, cardiovascular and respiratory mortality, but also for cancer-related mortality. Considering the log-linear effect, the OR for days with average temperature < =3rd percentile were significantly higher than the OR for days with average temperature < =5th percentile. The OR for days with average temperature < =1st percentile were significantly higher than the OR for days with average temperature < =3rd percentile and < =5th percentile (Table 1). In details, for days with average temperature < =5th percentile, considering log-linear effect, a 1 °C decrease in AWCET can result to OR for cancer-related mortality of 1.708 [1.665, 1.753], 1.803 [1.760, 1.848], 1.706 [1.659, 1.755], and 1.829 [1.780, 1.880], controlling for all factors. For days with average temperature < =3rd percentile, considering the log-linear effect, a 1 °C decrease in AWCET can result to OR for cancer-related mortality of 1.887 [1.813, 1.964], 2.020 [1.943, 2.101], 1.936 [1.854, 2.022], and 2.113 [2.025, 2.205]; and for days with average temperature < =1st percentile, a 1 °C decrease in AWCET can result to OR for cancer-related mortality of 2.337 [2.087, 2.615], 2.594 [2.318, 2.902], 2.404 [2.144, 2.697], and 2.845 [2.539, 3.187]. Similar patterns were also found for the results considering the curve-linear effect.

### Comparison between AWCET and NWCET

Based on the comparison of log-linear effect, this study has found that using AWCET can better address the magnitude of mortality risk for all perceivably cold days than using NWCET (Table 2).

**Table 2:** Odds ratios (OR) for comparison between AWCET and NWCET

Temperature thresholds for cases		Controls: (Same day and same weekday for 4 weeks before)		Controls: (Same day and same weekday for 8 weeks before)		Controls: (Same day and same weekday for 4 weeks after)		Controls: (Same day and same weekday for 8 weeks after)	
		AWCET	NWCET	AWCET	NWCET	AWCET	NWCET	AWCET	NWCET
All-cause mortality	Average temperature < = 5th percentile	**1.706 [1.682, 1.731]**	1.630 [1.608, 1.651]	**1.794 [1.770, 1.819]**	1.707 [1.685, 1.728]	**1.705 [1.679, 1.732]**	1.629 [1.605, 1.652]	**1.825 [1.798, 1.853]**	1.734 [1.710, 1.758]
	Average temperature < = 3rd percentile	**1.877 [1.837, 1.919]**	1.775 [1.740, 1.810]	**2.011 [1.968, 2.054]**	1.890 [1.854, 1.928]	**1.937 [1.892, 1.983]**	1.822 [1.783, 1.862]	**2.108 [2.059, 2.157]**	1.966 [1.925, 2.008]
	Average temperature < = 1st percentile	**2.463 [2.303, 2.634]**	2.211 [2.088, 2.341]	**2.768 [2.588, 2.960]**	2.457 [2.321, 2.601]	**2.514 [2.356, 2.683]**	2.261 [2.136, 2.393]	**2.947 [2.764, 3.142]**	2.617 [2.474, 2.768]
Cardiovascular mortality	Average temperature < = 5th percentile	**1.718 [1.668, 1.770]**	1.640 [1.596, 1.685]	**1.802 [1.752, 1.854]**	1.713 [1.669, 1.758]	**1.704 [1.650, 1.758]**	1.627 [1.580, 1.675]	**1.817 [1.762, 1.875]**	1.726 [1.677, 1.775]
	Average temperature < = 3rd percentile	**1.880 [1.798, 1.967]**	1.777 [1.706, 1.851]	**2.011 [1.924, 2.101]**	1.891 [1.817, 1.968]	**1.932 [1.841, 2.028]**	1.817 [1.739, 1.899]	**2.092 [1.995, 2.193]**	1.951 [1.870, 2.037]
	Average temperature < = 1st percentile	**2.595 [2.233, 3.015]**	2.311 [2.034, 2.625]	**2.959 [2.544, 3.440]**	2.602 [2.290, 2.956]	**2.564 [2.232, 2.946]**	2.296 [2.035, 2.590]	**2.961 [2.584, 3.393]**	2.623 [2.330, 2.954]
Respiratory mortality	Average temperature < = 5th percentile	**1.691 [1.642, 1.741]**	1.615 [1.573, 1.659]	**1.771 [1.723, 1.821]**	1.686 [1.644, 1.729]	**1.719 [1.665, 1.774]**	1.641 [1.594, 1.689]	**1.838 [1.782, 1.896]**	1.745 [1.696, 1.796]
	Average temperature < = 3rd percentile	**1.853 [1.773, 1.938]**	1.753 [1.683, 1.825]	**1.983 [1.898, 2.071]**	1.866 [1.793, 1.941]	**1.939 [1.849, 2.034]**	1.824 [1.746, 1.905]	**2.107 [2.010, 2.209]**	1.965 [1.883, 2.051]
	Average temperature < = 1st percentile	**2.438 [2.125, 2.798]**	2.191 [1.949, 2.464]	**2.756 [2.404, 3.160]**	2.449 [2.181, 2.751]	**2.501 [2.196, 2.849]**	2.256 [2.013, 2.528]	**2.945 [2.590, 3.350]**	2.622 [2.341, 2.937]
Cancer-related mortality	Average temperature < = 5th percentile	**1.708 [1.665, 1.753]**	1.631 [1.593, 1.670]	**1.803 [1.760, 1.848]**	1.715 [1.676, 1.754]	**1.706 [1.659, 1.755]**	1.630 [1.589, 1.672]	**1.829 [1.780, 1.880]**	1.737 [1.694, 1.781]
	Average temperature < = 3rd percentile	**1.887 [1.813, 1.964]**	1.783 [1.719, 1.849]	**2.020 [1.943, 2.101]**	1.899 [1.833, 1.968]	**1.936 [1.854, 2.022]**	1.821 [1.751, 1.895]	**2.113 [2.025, 2.205]**	1.971 [1.896, 2.048]
	Average temperature < = 1st percentile	**2.337 [2.087, 2.615]**	2.110 [1.917, 2.322]	**2.594 [2.318, 2.902]**	2.319 [2.109, 2.550]	**2.404 [2.144, 2.697]**	2.169 [1.962, 2.397]	**2.845 [2.539, 3.187]**	2.529 [2.290, 2.794]

OR with bold text indicates a result with significantly higher magnitude of mortality risk compared to another group of result

For all-cause mortality, 1 °C decrease in AWCET had 7.6–9.1% higher odds of mortality risk than 1 °C decrease in NWCET for days with average temperature < = 5th percentile, controlling for the effects of sociodemographic characteristics, neighbourhood disparity, urban/rural difference, air quality, and humidity as well as the weekday/weekend effect. The difference in magnitude of mortality risk was even stronger for colder days. For days with average temperature < = 3rd percentile and < = 1st percentile, 1 °C decrease in AWCET found 10.2–14.2% and 25.2–33.0% higher odds of mortality risk than 1 °C decrease in NWCET.

Similar evidences can be found for cause-specific mortality. For cardiovascular mortality, using AWCET could indicate 7.7–9.1%, 10.3–14.1%, and 26.8–35.7% higher odds of mortality risk than using NWCET for days with average temperature < = 5th percentile, <= 3rd percentile and < = 1st percentile, controlling for all factors. For respiratory mortality, the results for 1 °C decrease in AWCET had 7.6–9.3%, 10.0–14.2%, and 24.5–32.3% higher odds of mortality risk than the results for 1 °C decrease in NWCET for days with average temperature < = 5th percentile, <= 3rd percentile and < = 1st percentile. For cancer-related mortality, 1 °C decrease in AWCET had 7.6–9.2%, 10.4–14.2%, and 22.7–31.6% higher odds of mortality risk than 1 °C decrease in NWCET for days with average temperature < = 5th percentile, <= 3rd percentile and < = 1st percentile.

## Discussion

### Implications of cold mortality assessment

This study has found that AWCET is more appropriate to be used in a subtropical city. AWCET considers the wind chill effect in the subtropical context, which does not overstate the perception of wind on thermal comfort, but still consider a notable impact of wind loads that urban population should be aware of. Based on the validation, it is found that AWCET can indicate higher mortality on colder days. Specifically, this adjusted index with lower wind effect on thermal comfort could better demonstrate the difference in mortality between colder days and less cold days in Hong Kong, comparing to using traditional NWCET designed for temperate city. This is important, because our model has been controlled for the effects of sociodemographic characteristics, neighbourhood disparity, urban/rural difference, air quality and humidity, in which the validated results were highly based on the independent effect of difference in temperature.

Based on the results, we conclude that the use of AWCET should be recommended to the local government in a bottom-up context. This recommendation of using AWCET is also aligned with the action plan of local government agency. Based on the 5-items “Actions to be taken for cold weather warning” published by the Hong Kong Observatory (https://www.hko.gov.hk/wservice/warning/coldhot.htm), it is clearly stated that a person working outdoors during a day with cold weather warning should avoid prolonged exposure to wintry winds. As an additional strategy for the current action plans of the HKO, the use of AWCET is more suitable as a supplementary weather alert in district-level context. Based on the use of NET, HKO can identify significantly cold days for warning. However, NET itself compiled with multiple components of weather information, which may not be easily implemented to a district-level microclimate advisory system. Therefore, as a simplified version of biometeorological index, AWCET can provide an enhanced application for district-level monitoring, which can provide spatiotemporal information of weather measure with cold alerts to people in various locations. Such approach can also be aligned with the Community Weather Information Network (Co-Win) co-developed by the HKO and other agencies, in which this network has weather instruments covering urbanized and high-density environment of Hong Kong. In addition, even heat mortality is not as serious as cold mortality in Hong Kong, the Government has started to prepare more action plans for monitoring heat stress, including an enhancement of NET to a “Hong Kong Heat Index” for summer heat assessment [31]. This action partially addressed the needs of developing AWCET for enhancement of local cold warning system.

### Limitations and future directions

In this study, a limitation is that the mortality dataset of this study does not have linkage to any records of medical history for all decedents. It reduced the ability to including the pre-existing comorbidities in data modelling. However, since our focus was not to analyze cold effects on different causes of deaths; instead, this study aimed to evaluate whether AWCET is useful to predict mortality risk caused by known diseases associated with cold weather in Hong Kong such as cardiorespiratory diseases [7, 13, 14], our approach is therefore still appropriate.

Another limitation of this study is the statistical modelling itself, in which we considered a log-linear effect and curve-linear effect from temperature change, instead of a U-shape nonlinear function. This can be a limitation if this study was aiming to a perform a time-series analyses including both summer and winter mortality as part of the data analyses. However, since this study aimed to evaluate whether there was a sudden increase in mortality due to significantly drops in temperature within a short-period, and the change in mortality within this short period was generally more log-linear or curve-linear, the approach using a binomial regression without considering non-linear effect is still acceptable. More importantly, consideration of log-linear change in mortality caused by extreme temperature during a short period of time have been widely used in other studies [23–25]. Based on the stable results from all controls, it is also able to conclude that log-linear and curve-linear is appropriate in this research. In addition, a sensitivity test with adding cubed terms to the regression have also been applied to evaluate whether results will be modified by curvilinearity. Based on the sensitivity test, extreme cold events still had higher mortality risk than the other days based on the estimation with AWCET, and days with lower temperature (e.g. 1st percentile) have significantly higher mortality risk than days with relatively high temperature (e.g. 5th percentile).

In addition, this study focused on the temperature-mortality relationship to evaluate the use of AWCET and NWCET on cold mortality assessment in a subtropical city. However, consecutive cold days especially cold wave could also have a strong impact on mortality risk, instead of only the adverse impact from lower temperature. Specifically, previous studies have found that cold waves in Hong Kong could induce 3–4 weeks of elevated mortality risk [7, 14]. Therefore, it is recommended to include all definitions of cold wave with the use of AWCET to study the mortality displacement in Hong Kong, for a more comprehensive study. Along to this, some studies also suggested that public perceptions of extreme weather may have a higher contribution on health risk [32]. Therefore, a future study for cold wave should also be conducted by including information of subjective feelings and objective temperature measures (e.g. AWCET) for mortality assessment [33].

Furthermore, we used 1/3.6 as the adjustment of wind velocity based on the comments from local meteorological professionals. However, this application may not be robust and may involve with subjective bias. In order to justify this decision, a sensitivity analysis was conducted by comparing the results from the days with average temperature < =5th percentile with 1) the results using 1/2 and 1/3 as the adjustment of wind velocities, and 2) the results using ambient temperature without inclusion of wind velocity. Based on the sensitivity analysis, adjusted ORs estimated based on the model using 1/3.6 as the wind adjustment were higher than the other results. Therefore, the development of AWCET is still appropriate even it cooperated with subjective decisions from meteorological professionals.

Finally, bias from selection of control groups is a challenge for time-stratified study. Therefore, this study has followed previous research to use the multiple sets of controls and aims to reduce bias from selection of control groups [23].

## Conclusion

This study developed a modified version of wind chill index, namely “Adjusted Wind Chill Equivalent Temperature” (AWCET). AWCET was evaluated with the mortality data and was found to be useful for cold mortality assessment, specifically in the context of subtropical cities. The use of AWCET may be able to enhance the cold weather warning system in subtropical cities such as Hong Kong, as a supplementary tool to help demonstrating district-level perceived temperature [34] with the use of low-cost weather instrument from government-driven community network.

## Data Availability

All data generated or analyzed during this study are available from the corresponding author on reasonable request.

## Abbreviations

AWCET: Adjusted Wind Chill Equivalent Temperature
Co-Win: Community Weather Information Network
EPD: Environmental Protection Department
foreign languages %: percent of the population speaking foreign languages
HKO: Hong Kong Observatory
ICD-10: International Statistical Classification of Diseases and Related Health Problems
low education %: Percent of low-education population
NDVI: Normalized Difference Vegetation Index
NET: Net effective temperature
NO_X_: Nitrogen oxides
NWCET: New Wind Chill Equivalent Temperature
O_3_: ground-level ozone
OR: Odds ratio
PET: Physiological equivalent temperature
PM_2.5_: fine particulate matters
PM_10–2.5_: coarse particulate matters
RSP: Respirable suspended particulates
TPU: Tertiary planning unit
